# FRET-based carbazole-fluorescein ionic nanoparticle for use as an effective bioimaging agent

**DOI:** 10.55092/bm20230006

**Published:** 2023-07-21

**Authors:** Amanda Jalihal, Hannah Krehbiel, Samantha Macchi, Mavis Forson, Mujeebat Bashiru, Thuy Le, Caroline Kornelsen, Noureen Siraj

**Affiliations:** 1 Department of Chemistry, University of Arkansas at Little Rock, 2801 S. University Ave,. Little Rock, AR 72204, USA

**Keywords:** ionic material, FRET, bioimaging, fluorescent probe

## Abstract

Fӧrster resonance energy transfer (FRET)-based systems are widely applicable in many areas of interest. In this study, a novel FRET-based ionic material (IM) was synthesized by pairing carbazole imidazolium cation (CI^+^) with fluorescein anion (Fl^2−^) through a simple ion-exchange method. The resulting IM ([CI]_2_[Fl]) was converted into an ionic nanoparticle (INP) in aqueous media for practical use for bioimaging application. The photophysical properties of the parent dyes, [CI]_2_[Fl], and INP were studied in detail. All FRET parameters were calculated in the synthesized material. [CI]_2_[Fl] exhibited a significant spectral overlap integral and an ideal theoretical FRET distance. The presence of the FRET mechanism was verified by the observed decrease in donor fluorescence lifetime and a moderate FRET efficiency in [CI]_2_[Fl]. The INP formed from [CI]_2_[Fl] was evaluated for use as a fluorescent pH probe and bioimaging agent. FRET efficiency of INP is calculated in a series of pH studies which indicates the highest efficiency at physiological pH. Whereas no FRET phenomenon is observed in highly acidic and basic conditions. The pH-dependent photophysical properties of [CI]_2_[Fl] are monitored and allow for the potential application as a fluorescent probe for the detection of acidic tissues in biological systems. The FRET-capable INP showed superior bioimaging capability *in vitro* as compared to the parent dye.

## 1. Introduction

Förster Resonance Energy Transfer (FRET) imaging is widely used in various applications such as detection of biomarkers [1], protein interaction studies [2], drug release monitoring [3], and bioimaging [4]. This mechanism of nonradiative energy transfer occurs between a donor fluorophore and an acceptor chromophore [5,6]. For this process to occur, certain criteria are required. First, a significant overlap between the fluorescence emission of the donor and the absorbance spectra of the acceptor must be observed [2]. This overlap provides the possibility for the donor to pass energy to the acceptor [7]. The second criterion is that the donor and acceptor must be within a distance of 10 nm [8]. The separation of the two moieties beyond this distance will inhibit the transfer of energy from the donor to the acceptor [6]. FRET-based imaging is ideal for bioimaging purposes. This technique is non-invasive, non-destructive [3], and has low detection limits [4]. Incorporating FRET mechanism in a bioimaging probe, it enhances the spectral gap between excitation and emission, as compared to a single fluorophore, which reduces background noise while imaging [9].

FRET-active molecules can also be employed as fluorescent probes. Fluorescent probes differ from bioimaging agents in that they detect specific proteins, enzymes, or tissues by observable changes induced in fluorescent properties after contact with the said targets of interest [12]. Two basic categories of fluorescent probes are intensity-based and ratiometric. Intensity-based probes are limited due to variations in fluorescence intensity caused by their surrounding environment [10]. Ratiometric fluorescent probes resolve this issue by including a built-in background correction that measures fluorescence emission intensity at two separate wavelengths. The ratio of the emissions gives a signal independent of environmental factors and allows for quantitative measurement of intensity [10–12]. Current ratiometric probe techniques include block polymer encapsulated donor-acceptor pairs [3], colorimetric chemosensors [13], excited-state intramolecular proton transfer probes [14], photoacoustic probes [15], fluorescent nanodiamonds [16], quantum dots [17], and nanoparticles (NPs) [18].

To date, NPs have shown great potential for bioimaging and fluorescent probe applications. The size, surface functionalities, and morphology of NPs can be tuned to fit specific task making them widely useful [19]. NPs have the added benefit of enhanced cellular uptake which was observed in previous studies due to enhance permeability and retention effect (EPR) of NPs. [20,21]. FRET-based NPs have been successfully reported but limited due to biocompatibility issues [22,23]. To enhance the biocompatibility of the NPs, nanovesicles have been prepared using liposomes or polymeric micelles as carriers. These NPs are carriers for hydrophobic and hydrophilic bioimaging agents in biological systems; however, they suffer from aggregation and short shelf life [24]. Thus, a biocompatible, shelf-stable and carrier free nanomaterial is needed for biological applications.

Fluorescein presents a potentially safe option for such applications. The dye has been investigated previously as a bioimaging agent and fluorescent probe due to its high quantum yield, strong absorptivity, selectivity, and low toxicity [4,25–27]. Fluorescein and its derivatives have been extensively explored for bioimaging purposes [28–30]. The photophysical properties of fluorescein are highly pH-dependent thus indicating the potential for a pH-dependent fluorescent probe. Fluorescein can exist as a dianion, monoanionic, and neutral molecule with pKas of 6.3, 4.3, and 2.2, respectively [31]. These different forms of fluorescein have unique absorbance and fluorescence emission spectra. The absorbance wavelength maximum of each form differs by at least 20 nm [27]. The fluorescence emission intensity is strong in the dianion form and significantly reduced in the monoanionic form. The neutral form of the molecule has no fluorescent properties [31]. This unique property of fluoresceine opens the possibility for its use as a biocompatible, pH-dependent fluorescent probe [32].

In this work, an ionic material (IM) is investigated for use as a FRET-based bioimaging agent and fluorescent probe. The possibility of the FRET mechanism in an IM has been supported in a previous study [33]. This novel FRET-based IM is designed using carbazole imidazolium (CI^+^) as the donor moiety and fluorescein (Fl^2−^) as the acceptor. CI^+^ was selected as the donor moiety because carbazole derivatives are known to act as good electronic donators and exhibit excellent planarity, biocompatibility, and photostability [34–36]. CI^+^ and Fl^2−^ demonstrate significant spectral overlap between CI^+^ fluorescence emission and Fl^2−^ absorbance, thus meeting the first criterion for the FRET mechanism. The conversion of the parent dyes into the IM ([CI]_2_[Fl]) is a simple ion-exchange reaction that produced ionic dyes (CI^+^ and Fl^2−^) while removing the spectator ions (I^−^ and Na^+^) [33]. The absence of spectator ions allows the dye ions to come in closer proximity, falling within the 10 nm FRET distance requirement [33,37]. These properties provide sufficient evidence that the FRET mechanism can occur between CI^+^ and Fl^2−^. Additionally, ionic nanoparticles (INPs) are prepared from the FRET-based IM in aqueous media for practical use in a biological system [38]. Confocal microscopy was used to validate the bioimaging potential of the INP for use *in vitro* and pH-dependent studies were conducted to verify the capacity for fluorescent probe application.

## 2. Methods

### 2.1. Material

CII was synthesized similarly as reported in a previous study [24,28,29]. Sodium fluorescein was purchased from Mallinckrodt, Inc (St. Louis, MO). Ethanol, hydrochloric acid (HCl), sodium hydroxide (NaOH), and dichloromethane (DCM) were purchased from VWR (Radanar, PA). MCF-7 model cell line was purchased from ATCC^®^ (HTB-22) (Manassas, VA). Dulbecco’s Modified Eagle Media (DMEM), fetal bovine serum (FBS), penicillin/streptomycin, acetic acid, dibasic sodium phosphate, coverslips (12 mm borosilicate glass), Permount^™^ mounting media, and tissue culture plates were purchased from Fisher Scientific (Walthram, MA). Paraformaldehyde and MitoRed were purchased from Santa Cruz. Phosphate buffered saline (PBS, pH 7.4) was purchased from ThermoFisher (Walthram, MA). Glycine was purchased from Eastman Organic (Kingsport, TN). Sodium acetate was purchased from Fluka (Walthram, MA). Citric acid was purchased from Alfa Aesar (Haverhill, MA). Distilled deionized water of 18.2 MΩ cm was obtained from ultrapure distilled water purifier (ELGA).

### 2.2. Synthesis of [CI]_2_[Fl] and INPs

**Figure 1.**
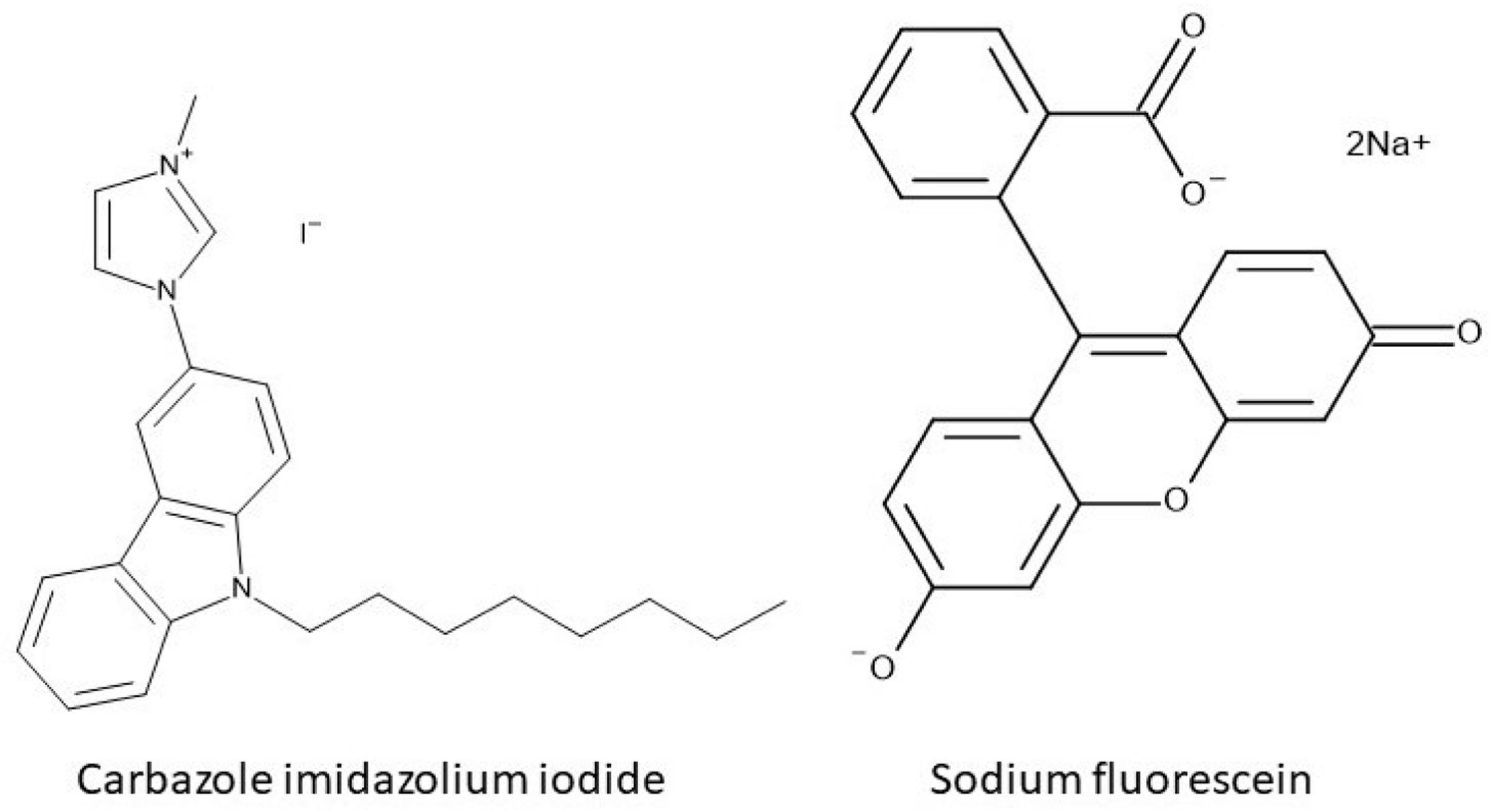
Structure of parent compounds CII and Na2Fl.

[CI]_2_[Fl] was synthesized by a previously reported ion exchange method [41–43]. Briefly, Na_2_Fl was dissolved in water, and two molar equivalents of CII were dissolved in DCM at roughly 5 times the volume. The structure of parent compounds, CII and Na_2_Fl are shown in Figure 1. These two immiscible solutions were combined and stirred for 48 hours. Afterward, the aqueous layer, containing the spectator ions (Na^+^ and I^−^) was extracted and discarded. The DCM layer, containing the [CI]_2_[Fl] product, was washed with water to remove any residual spectator ions impurities from the parent compounds. The DCM was evaporated under reduced pressure using rotary evaporation to yield a solid [CI]_2_[Fl]. [CI]_2_[Fl] arranges in a two CI^+^ to one Fl^2−^ ratio, as noted in Equation 1. The inclusion of both ions in [CI]_2_[Fl] was confirmed by mass spectrometry using a Shimadzu IT-TOF ESI high-resolution mass spectrometer. As shown in Figure S1, the observed peaks of the positive and negative ion modes were 360.2434 and 333.0757, respectively. These values were consistent with the calculated molecular weight of CI^+^ ion (360.51 g/mol) and Fl^2−^ ion (330.29 /mol). Fragments were verified by comparing the mass to charge ratio of the ions.

(1) $$Na_{2}Fl_{aq}+2CII_{org}\to {\left[CI\right]}_{2}{\left[Fl\right]}_{org}+2NaI_{aq}$$

The INP of [CI]_2_[Fl] was prepared through a reprecipitation method [31]. [CI]_2_[Fl] was dissolved in ethanol at a high concentration. A small volume of [CI]_2_[Fl] solution was added to 5 mL of water under bath sonicator and subjected to sonication waves for 5 minutes. After agitation, the mixture was rested for 20 minutes to reach colloidal stability. The resultant sample contained [CI]_2_[Fl] dispersed in aqueous media in the INP form.

### 2.3. Materials characterization

Thermogravimetric analysis (TGA) was performed using a Mettler Toledo instrument to verify the thermal stability of the solid [CI]_2_[Fl]. Samples were heated in air at a rate of 10 °C per minute from 25–800 °C. The INP size was characterized by dynamic light scattering (DLS).

### 2.4. Photophysical studies

The absorption spectra of CII, Na_2_Fl, [CI]_2_[Fl], and INP were recorded using a Cary 60 UV-Vis Spectrophotometer. The absorbances of the samples were measured in a 10 mm path length, polished two-sided quartz cuvette (Starna Cells), and measured against an identical cell filled with the respective solvent.

Fluorescence emission of all samples was recorded using a Horiba FluoroMax Fluorescence Spectrophotometer. A 10 mm path length, polished four-sided quartz cuvette (Starna Cells) was used for fluorescence emission measurements. All samples were recorded using the same slit width, the integration time of 0.1 seconds, and at right angle geometry.

### 2.5. FRET calculations

The spectral data was used to calculate the spectral overlap integral (J(λ)) and Förster distance (${\text{R}}_{\text{o}}$) using Equation 2 and 3, respectively.

(2) $$J(\lambda )=\frac{\int_{350}^{545}\varepsilon (\lambda )f(\lambda )\lambda^{4}d\lambda }{\int_{350}^{545}f(\lambda )d\lambda }$$

Where ε(λ) is the molar extinction coefficient (M^−1^cm^−1^) of Na_2_Fl at the overlap wavelength λ and f(λ) is the normalized fluorescence intensity of CII when excited at 277 nm.

(3) $$R_{0}=0.0211{\left(\frac{k^{2}\phi_{D}J}{n^{4}}\right)}^{1/6}$$

Where $k^{2}$ is the dipole orientation factor (conventionally assumed to be 2/3) [8], $\phi_{D}$ is the quantum yield of the CII previously determined as 25% [39], J is the spectral overlap integral, and 𝑛 is the refractive index of the medium.

The relative quantum yield of the donor and acceptor in [CI]_2_[Fl] was calculated using the relative quantum yield method, Equation 4.

(4) $${\text{Φ}}_{un}={\text{Φ}}_{s}\left(\frac{I_{un}}{I_{s}}\right)\left(\frac{Abs_{s}}{Abs_{un}}\right)\left(\frac{\eta_{un}}{\eta_{s}}\right)$$

Where Φ is the quantum yield of the unknown (un) and standard (s), I is the integrated intensity, Abs is the absorbance at the excitation wavelength, and η is the refractive index of the media [44]. The reported quantum yields of 25% for CII and 95% for Na_2_Fl were used as the standard values in the calculation for donor and acceptor, respectively [39,45].

The FRET energy transfer efficiency between CI^+^ and Fl^2−^ was determined using Equation 5.

(5) $$E\text{\%}=\left(1-\frac{F_{da}}{F_{d}}\right)\times 100$$

Where $F_{da}$ is the fluorescence emission intensity of CI^+^ in the presence of Fl^2−^ and $F_{d}$ is the fluorescence emission intensity of CI^+^ in the absence of the acceptor [37].

Fluorescence lifetimes of the donor moiety in the parent dye (CII) and [CI]_2_[Fl] were determined using Horiba NanoLED Pulsed Diode Controller with a NanoLED N-270 nm excitation source. The instrumental response is analyzed using DOS6 software.

### 2.6. pH studies

To study the effect of pH on FRET efficiency, INPs’ spectral properties were evaluated in a range of pH buffers from 2–11. Individual aqueous buffers (described in SI) were prepared and adjusted to the desired pH with HCl and NaOH. INPs of [CI]_2_[Fl] was prepared in each solution as previously described. The absorbance and fluorescence emission intensity were recorded at a respective wavelength maxima for different pHs. And the FRET efficiency of [CI]_2_[Fl] was also calculated at each pH.

### 2.7. Confocal fluorescence microscopy

In *vitro* confocal fluorescence microscopy experiment was designed to evaluate the bioimaging potential of INPs. MCF-7 cells were maintained as a monolayer at 37 °C and 5% CO_2_ in a complete medium. Cells were cultured in DMEM, with phenol red, supplemented with FBS (10% v/v) and penicillin/streptomycin antibiotic solution (500 units/ml).

Sample preparation was performed by plating approximately 1.2 × 10^5^ cells per well in a 24-well plate with coverslips placed on the bottom of the wells. Cells were allowed to attach onto coverslips for 24 hrs. Then [CI]_2_[Fl] or Na_2_Fl (5 μM) were incubated for 1 hr at 37 °C. Cells were washed three times with PBS to remove any free dye/particles. Then, cells were incubated with 150 nM MitoRed solution in PBS for 45 minutes, washed three times, and fixed with 200 μL paraformaldehyde (4%) for 15 minutes at room temperature. Slips were washed thrice with PBS and then mounted onto microscope slides with a small volume of mounting media. Confocal imaging was performed using a laser scanning confocal microscope (Zeiss, LSM 880), which was attached to an inverted microscope. An oil immersion objective lens (63X) was used for cell imaging. For confocal imaging, two channels were used: one using a 488 nm diode laser as the excitation source with spectrally tunable emission filter set at 515 nm for Fl^2−^ and the second using 561 nm excitation laser with emission at 594 nm for MitoRed stain.

## 3. Results

### 3.1. Materials characterization

The thermal stability of the solid [CI]_2_[Fl] was evaluated using TGA analysis. Both parent dyes and [CI]_2_[Fl] were heated under continuous airflow from 25–800 °C, shown in Figure S2. Na_2_Fl shows significant weight loss around 280 °C and gradually degrades until ~4000 °C; whereas CII remains stable up to 1150 °C but completely degrades at higher temperatures. [CI]_2_[Fl] retains thermal stability until 1150 °C and does not completely degrade until ~3000 °C.

### 3.2. Photophysical properties

**Figure 2.**
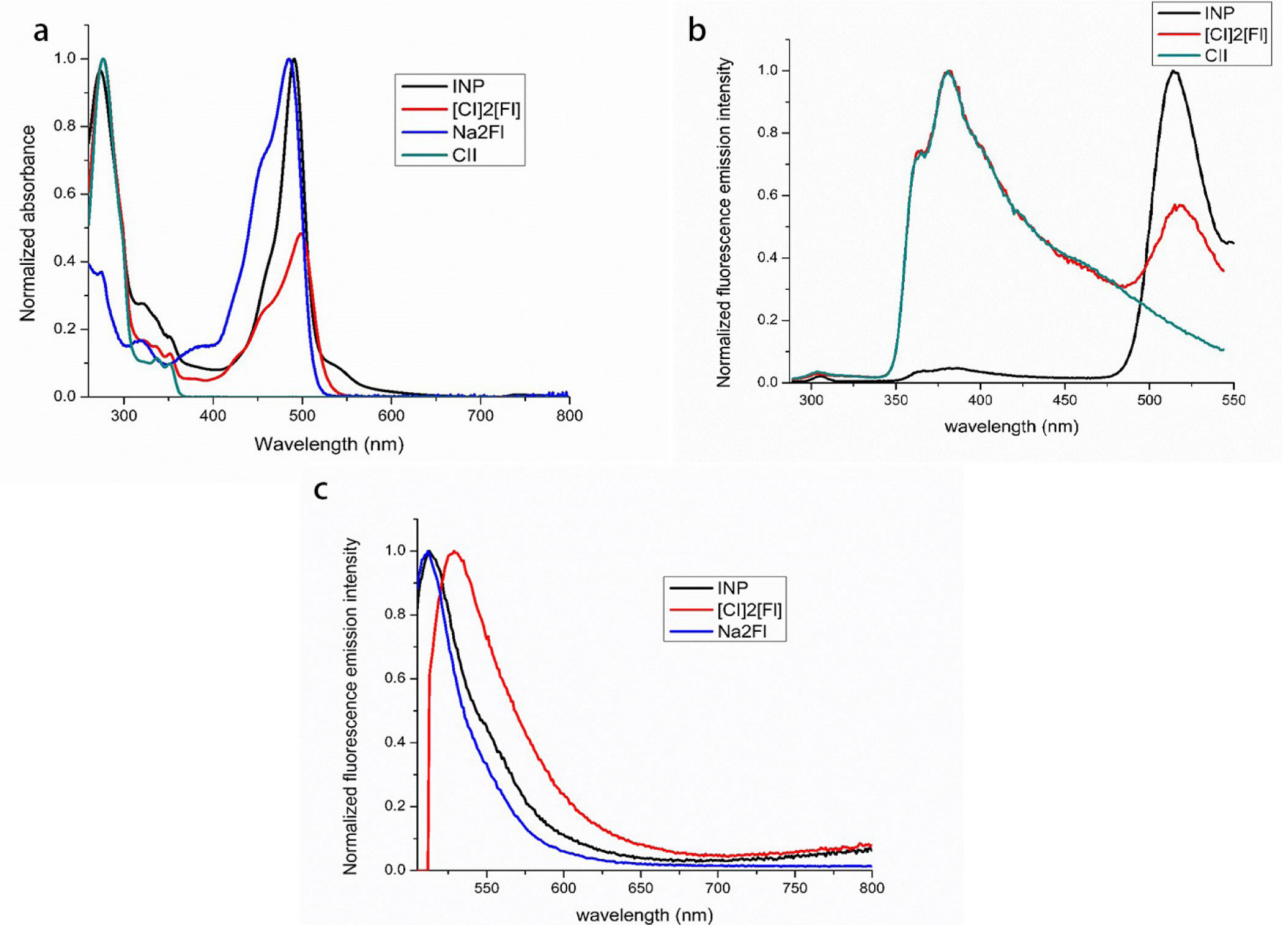
Normalized absorbance spectra of Na_2_Fl (normalized to 493 nm, water), CII (normalized to 277 nm, ethanol), [CI]_2_[Fl] (ethanol), and INP (water) (A), normalized fluorescence emission spectra of CII (normalized to 378 nm, ethanol), [CI]_2_[Fl] (normalized to 378 nm, ethanol), and INP (normalized to 511 nm, water) excited at 277 nm (B), and normalized fluorescence emission spectra of Na_2_Fl (normalized to 511 nm, water), [CI]_2_[Fl] (normalized to 525 nm, ethanol), and INP (normalized to 511 nm, water) excited at 493 nm (C).

Na_2_Fl, CII, [CI]_2_[Fl], and INP were characterized in detail using absorbance and fluorescence spectroscopy. The normalized absorbance and emission spectra of the samples are presented in Figure 2.

As demonstrated in Figure 2a, the absorption wavelength maxima of Na_2_Fl and CII were observed at 490 and 277 nm, respectively. Both peaks were present in [CI]_2_[Fl] and INP absorbance spectra confirming that the synthesized IMs contained both ions. Upon excitation at 277 nm (Figure 2b), the fluorescence emission wavelength maximum of the donor (CI^+^) was found to be 380 nm. [CI]_2_[Fl] and the INPs exhibit an emission peak at 380 nm when excited at 277 nm as well as an additional peak at 511 nm. The peak at 511 nm was assigned to fluorescein anion while the high energy transition peak was attributed to carbazole cation present in the [CI]_2_[Fl] and INPs. The later peak (511 nm) was not observed in the CII fluorescence emission spectra when excited at 277 nm nor was any emission observed for Na_2_Fl when excited at 277 nm, Figure S3. Thus, it is concluded that the energy transfer mechanism is present in the newly developed IM, [CI]_2_[Fl], and INPs as shown in Figure 2b. These interesting results led us to investigate the FRET parameters in [CI]_2_[Fl] where CI^+^ is acting as a donor while Fl^2−^ is serving as an acceptor. When comparing the fluorescence emissions of the INP and [CI]_2_[Fl], the fluorescence emission attributed to the donor (380 nm) is significantly reduced while the acceptor fluorescence emission (511 nm) is increased in the INPs, as shown in Figure 2b. This finding indicates that INP formation significantly enhances the spectral resolution of [CI]_2_[Fl] in that the INP can be excited at donor absorption maximum and fluorescence emission clearly recorded at the lower energy acceptor wavelength.

Na_2_Fl exhibited an absorption wavelength maximum of 493 nm (Figure 2a). When excited at 493 nm, the fluorescence emission wavelength maximum for Fl^2−^ was confirmed at 511 nm, noted in Figure 2c. [CI]_2_[Fl] and INP show a similar peak position to Na_2_Fl when excited at 493 nm thus indicating there were no structural changes in the fluorescein moiety in the [CI]_2_[Fl] or INP.

### 3.3. FRET calculations

The fluorescence emission spectrum of the donor (CII) and the absorbance spectrum of the acceptor (Na_2_Fl) was used to calculate the spectral overlap integral (J(λ)) and theoretical FRET distance (R0) for the ion pair. For energy transfer to take place, there must be sufficient overlap between the donor fluorescence emission and acceptor absorbance [46]. The spectral overlap integral value was determined using Equation 2 for CI^+^ and Fl^2−^ as 3.35×10^14^ M^−1^cm^−1^nm^4^. This value is sufficient and leads to the conclusion that the FRET mechanism is possible between the two ionic moieties.

The donor and acceptor must be within 10 nm of each other as well. Using Equation 3, the theoretical FRET distance of the CI^+^ and Fl^2−^ was calculated as 3.41 nm. Thus, these results along with the observed fluorescence emission at acceptor wavelength provide strong evidence that energy transfer is occurring between CI^+^ and Fl^2−^ [37][43].

The relative quantum yields for the donor and acceptor were calculated for [CI]_2_[Fl] as compared to the respective parent dye. The quantum yield of the donor (CI^+^) and acceptor (Fl^2−^) moieties were determined as 22.24% (excited at 277 nm) and 97.24% (excited at 493 nm) for [CI]_2_[Fl], respectively. As expected, the donor quantum yield in [CI]_2_[Fl] is slightly decreased as compared to the reported value of 25% for CII [28] however the parent compound contains the iodide anion, a well-known quencher. This reduced quantum yield value also validates the FRET mechanism in [CI]_2_[Fl] due to the energy being passed to the acceptor non-radiatively as opposed to emitted photon by donor cation. Whereas, the quantum yield of the acceptor is increased as compared to the reported value of 95% for Na_2_Fl [45] due to the energy received from the donor through the FRET mechanism.

The FRET mechanism is further verified by performing fluorescence lifetime measurements. Fluorescence lifetimes are measured by inducing an ultrashort pulse of light at the donor excitation wavelength (277 nm). The photon emission distribution after excitation is measured at the nanosecond scale and any decrease of the donor fluorescence emission results in a shorter lifetime. Presence of the FRET mechanism results in reduced donor fluorescence emission; therefore, the donor will have a shorter fluorescence lifetime if FRET is occurring [35]. Fluorescence lifetime for CII and [CI]_2_[Fl] are reported in Table 1. After the conversion of the parent CII compound into [CI]_2_[Fl], a reduction in fluorescence lifetime is observed from 5.80 ns to 5.49 ns, respectively.

After validation that the FRET mechanism is present between the two ionic moieties in [CI]_2_[Fl] and INP, the energy transfer efficiency was quantified using Equation 5. The FRET energy transfer efficiency for this donor-acceptor pair as an IM is calculated to be 27.10%. This value is not remarkably high; however, the FRET energy transfer efficiency is increased to 42.93% upon conversion into the INP. This finding indicates that the photophysical properties of the material are indeed improved in the INP form not only in the enhancement of the spectral resolution but in the FRET efficiency as well [43].

### 3.4. pH studies

**Figure 3.**
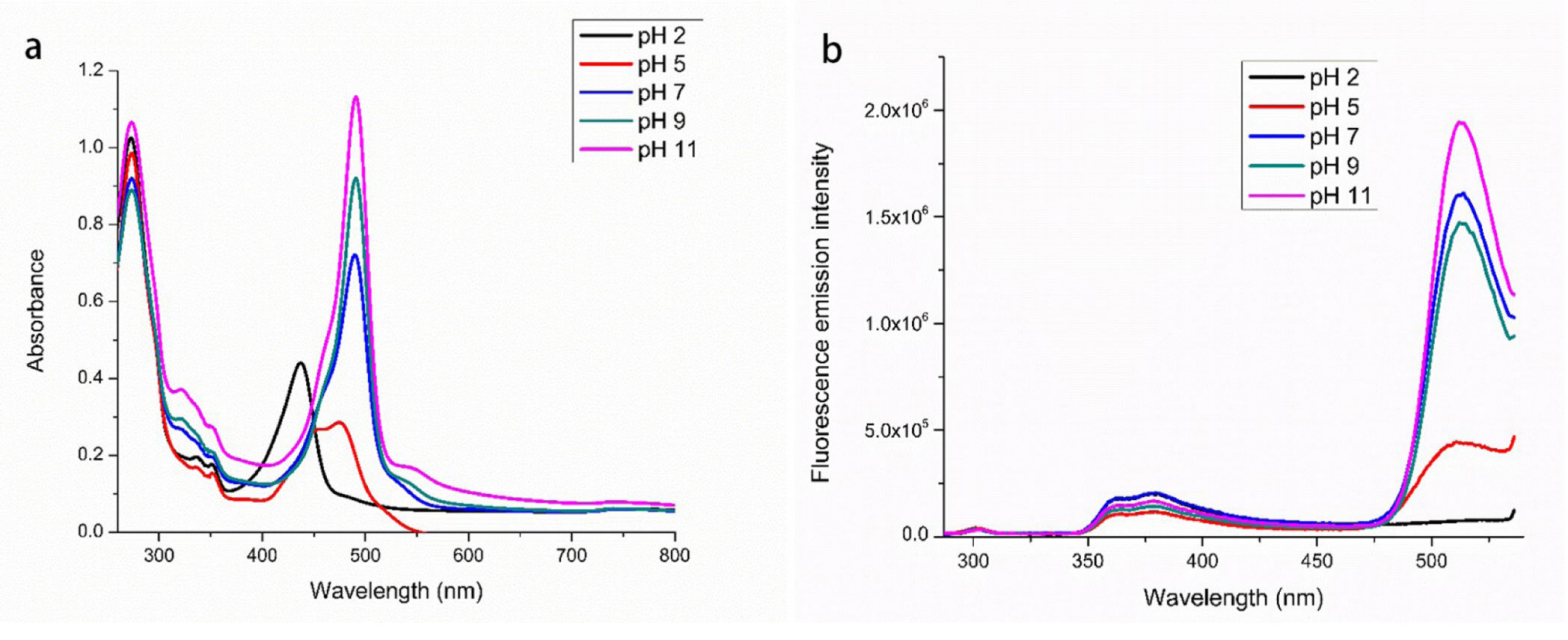
Absorbance (a) and fluorescence emission intensity excited at 277 nm (b) of INPs in various pH solutions.

The effect of pH on the FRET efficiency was studied in the INPs. INPs were prepared in pH buffers ranging from pH of 2–11. The absorbance and fluorescence emission spectra of each solution were recorded and are reported in Figure 3. In the absorbance spectrum (Figure 3a), pH 2 and 5 show the characteristic peaks for neutral and monoanionic fluorescein, respectively. The absorbance of fluoresceine at pH 2 and 5 is greatly reduced as well as slightly blue-shifted. This observation is expected and matches with the reported fluorescein pKa values of 2.2 and 6.7 [27]. At pH 7, 9, and 11 the absorbance of the Fl^2−^ acceptor increases with increasing pH. There is no significant change in the absorbance of the donor CI^+^ moiety observed at different pHs.

In Figure 3b, the fluorescence emission intensity for Fl^2−^ is significantly reduced in the pH 5 solution and not present in the pH 2 samples when excited at the donor excitation (277 nm). Since the pka value of fluoresceine suggest that fluorescence is present as a monoanionic form at pH 5 thus only one CI^+^ is paired with monoanion fluorescein and causing almost half energy transfer efficiency. At pH 2, fluorescein is present as a neutral specie thus there is no pairing with any CI^+^ donor and thus no peak was observed for fluorescein due to absence of energy transfer. Moreover, neutral fluorescein does not exhibit any fluorescent properties at lower pH [31]. The donor fluorescence emission intensity in the INP was compared to CII fluorescence emission to calculate the FRET efficiency at differing pH and is reported in Table 2. The fluorescence emission spectrum for CII in the pH solutions is presented in Figure S4.

Interestingly, the FRET energy transfer is most efficient at the physiological pH range. The higher FRET efficiency indicates that more energy is transferred from the donor (CI^+^) to the acceptor (Fl^2−^). The increased energy transfer will lead to an increase in the quantum yield of the acceptor and higher fluorescence emission intensity as indicated in Figure 3b. On the other hand, the FRET efficiency is quite low at pH 5. At the lower pH, the monoprotonated fluorescein acceptor will only interact with one CI^+^ thus reducing the potential for energy transfer. The unique property opens the possibility that the INPs could be used as a fluorescent probe for the detection of acidic tissues such as tumors or bacterial infections. The change in emission properties of a ratiometric probe at different pHs provide a tremendous benefit to use this material for wide range of application including biological application.

### 3.5. Confocal fluorescence microscopy

**Figure 4.**
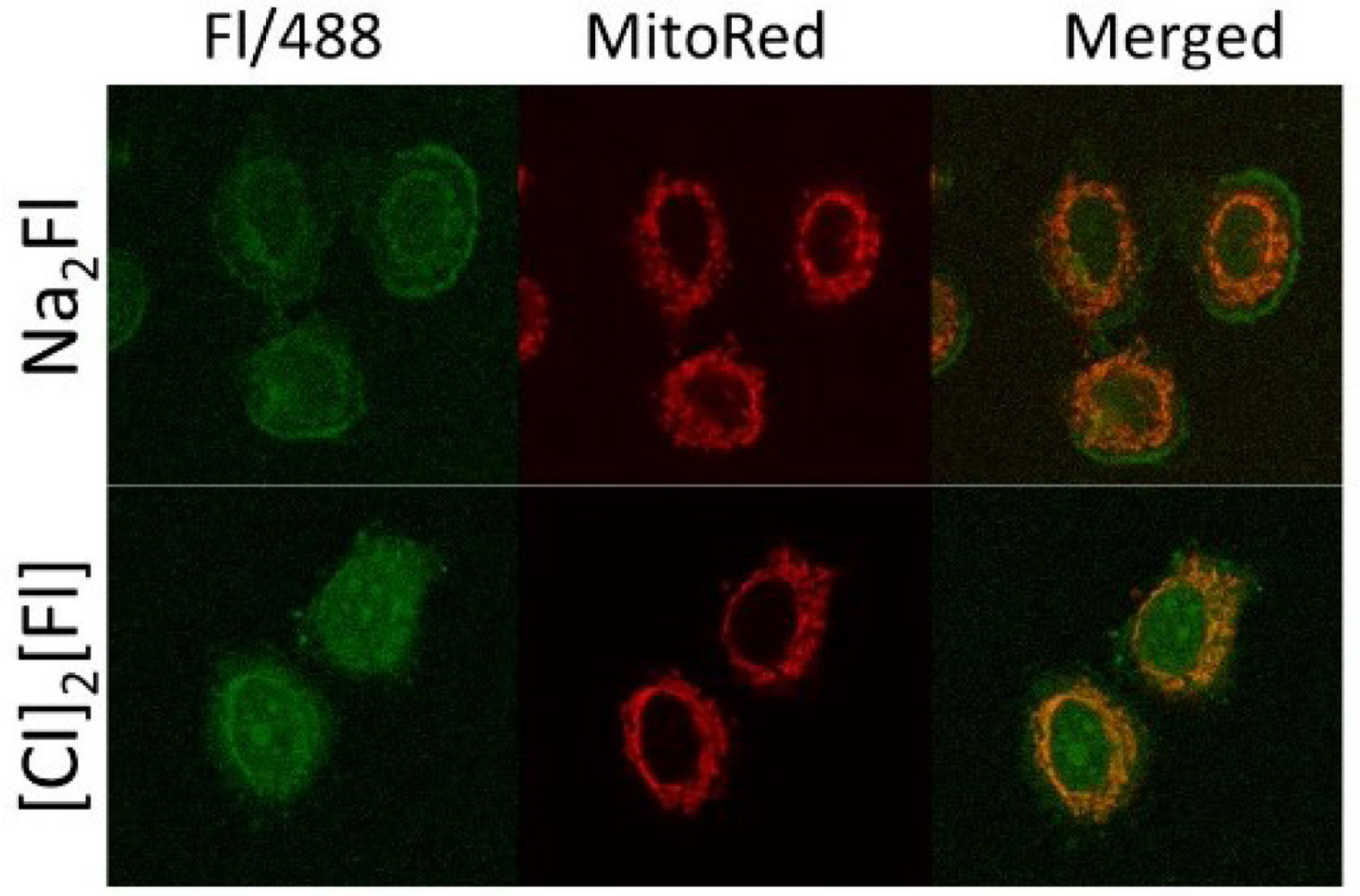
Confocal images of MCF-7 cells were incubated in 5 μM samples for 1 hour, stained with MitoRed, and fixated with paraformaldehyde.

Confocal microscopy experiment was designed to investigate the uptake and emission properties of INP in vitro. INPs have previously demonstrated superior cellular uptake in cancer cells when compared to the soluble parent drug [48,49]. As expected, when Na_2_Fl and [CI]_2_[Fl] were incubated with MCF-7 cells, the INP showed better uptake as compared to the parent dye. The improved uptake is due to the well-known enhanced permeability and retention (EPR) effect in tumor. The “leaky” nature of cancer cells allows the INPs to accumulate easily and the superior uptake accounts for enhanced bioimaging capability [50]. In this imaging experiment, MitoRed is used to stain the mitochondria of the cell to give a reference for the uptake location of the bioimaging agent. As seen in Figure 4, the Fl^2−^ emission (488 nm Ex/515 nm Em) is observed in both the soluble form (Na_2_Fl) and INP form ([CI]_2_[Fl]) at the same fluorophore concentration. However, a higher Fl^2−^ fluorescence intensity was observed in the INP-incubated sample. Moreover, the INP appeared to be located inside the mitochondria, indicating INP’s localization within the mitochondria. In comparison to the Na_2_Fl, Fl^2−^ fluorescence emission is located outside the mitochondrial barrier meaning that the uptake and subcellular localization of the Fl^2−^ is significantly impacted by converting soluble Na_2_Fl into INP. Thus, leading to the conclusion of enhanced uptake of the INP as compared to the soluble parent dye. This result provides strong evidence that the [CI]_2_[Fl] could be used as an effective bioimaging agent due to their improved quantum yield and enhanced cellular uptake. Moreover, the ratiomatric fluorescence response makes these INP ideal for bioimaging application.

## 4. Conclusion

A novel organic IM composed of CI^+^ cation and Fl^2−^ anion for use as a bioimaging agent ratiometric fluorescent pH probe was synthesized through a simple ion-exchange protocol. Spectral characteristics and photophysical calculations for the compound supported the idea that the FRET mechanism is possible between CI^+^ donor and Fl^2−^ acceptor pair. This was confirmed by the quantum yield of the donor and acceptor in [CI]_2_[Fl], the fluorescent lifetime of the donor, and the FRET efficiency of [CI]_2_[Fl] and INPs. Upon conversion into the INP, the spectral resolution and FRET efficiency are enhanced which suitable for use in a biological system. The INP shows excellent potential as a fluorescent probe as the emission significantly increased at the normal physiological pH. The INP also shows superior uptake and altered subcellular localization in vitro when compared to the soluble Na_2_Fl parent dye due to EPR effect. The improved quantum yield, ratiometric fluorescence and enhanced cellular uptake of FRET exhibiting [CI]_2_[Fl] INP makes it ideal candidate as bioimaging agent and fluorescent pH probe.

## Supplementary Material

Supplementary Information

## Figures and Tables

**Table 1.** Fluorescent quantum yield and fluorescent lifetime of CII and [CI]_2_[FL]

Sample	${\text{Φ}}_{\text{f}}$ at 277 nm (%)	τ (ns)

CII	25	5.80
[CI]_2_[Fl]	23.5	5.49

**Table 2.** FRET efficiency (%) of INPs at pH 2, 5, 7, 9, and 11.

Solution pH	FRET efficiency (%)

1.98	--
5.01	9.20
7.01	42.93
9.00	27.25
11.02	--
