# Supplementary material for: FRET-based carbazole-fluorescein ionic nanoparticle for use as an effective bioimaging agent
Source: Biofunctional Mater. Author manuscript; Available in PMC 2024 Jan 3. (PMC10763496; doi:10.55092/bm20230006)
Supplement: Supplementary Information [file NIHMS1948077-supplement-Supplementary_Information.pdf]

Article | Received 2 May 2023; Accepted 25 June 2023; Published 21 July 2023
https://doi.org/10.55092/bm20230006

Supplementary Data

FRET-based carbazole-fluorescein ionic nanoparticle for use as an effective bioimaging agent

Amanda Jalihal[1], Hannah Krehbiel[1], Samantha Macchi[1], Mavis Forson[1], Mujeebat Bashiru[1], Thuy Le[1], Caroline Kornelsen[1], and Noureen Siraj[1,*]

Department of Chemistry, University of Arkansas at Little Rock, 2801 S. University Ave,. Little Rock, AR 72204, USA

* Corresponding author; E-mail: nxsiraj@ualr.edu.

Supplementary material

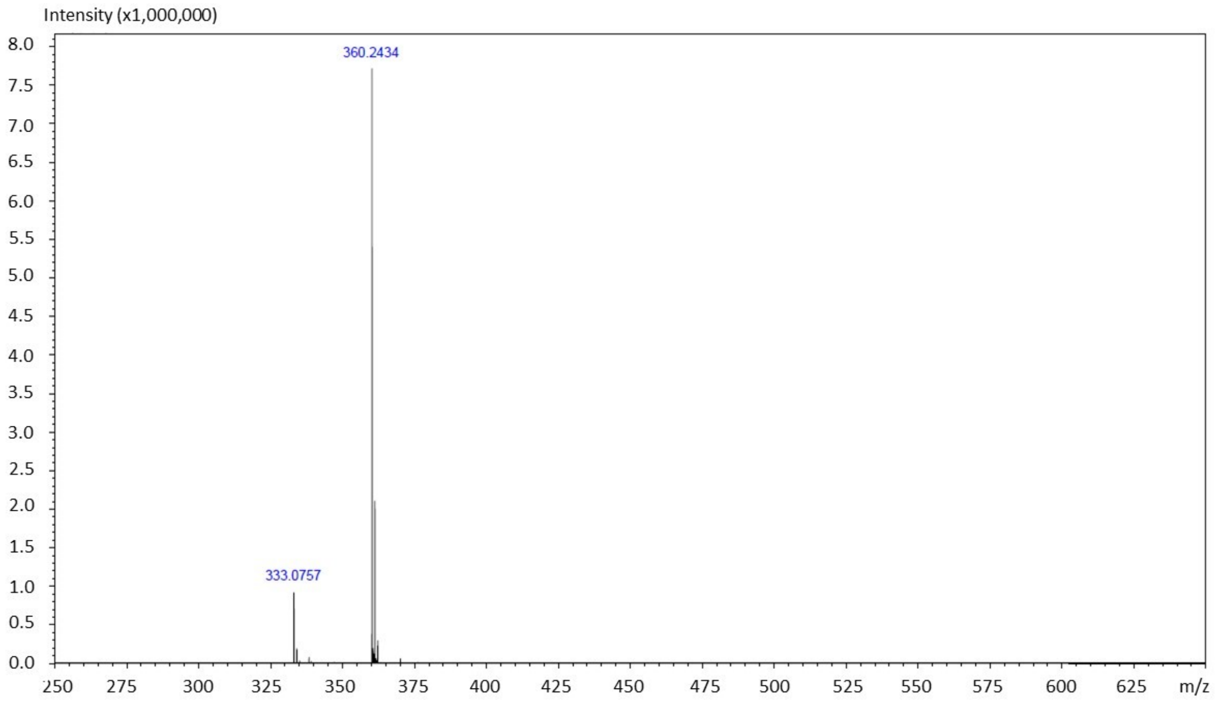

Figure S1. the observed peaks of the positive and negative ion modes were 360.2434 and 333.0757, respectively. These values were consistent with the calculated molecular weight of CI^{+} ion (360.51 g/mol) and Fl^{2-} ion (330.29/mol).

Copyright©2023 by the authors. Published by ELS Publishing. This work is licensed under Creative Commons Attribution 4.0 International License, which permits unrestricted use, distribution, and reproduction in any medium provided the original work is properly cited.

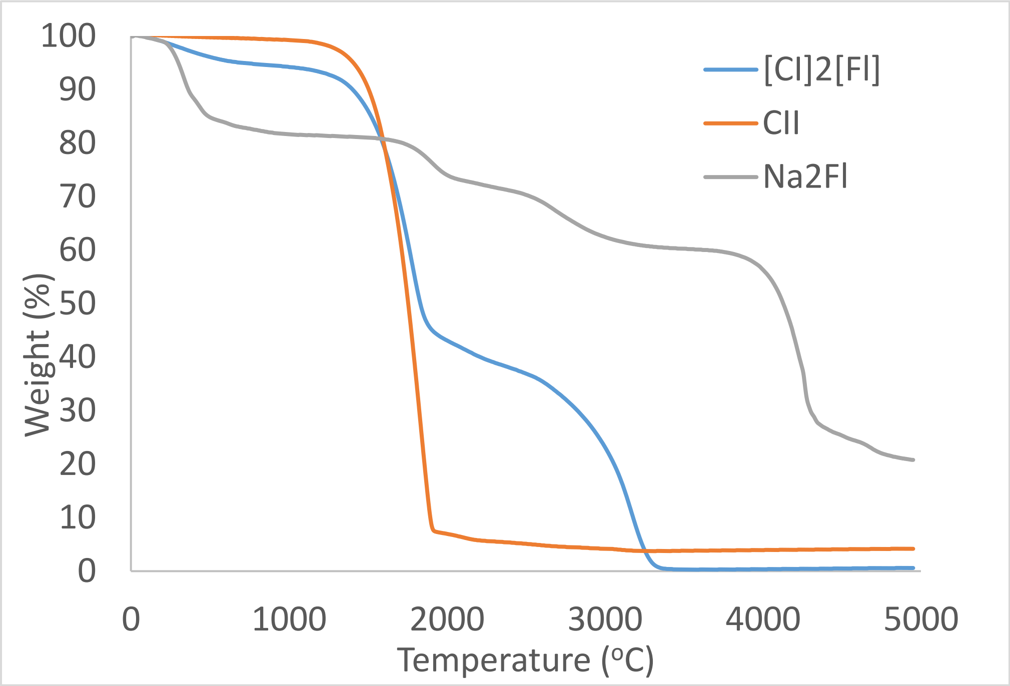

Figure S2. Both parent dyes and the IM were heated under continuous airflow from 25-800 °C.

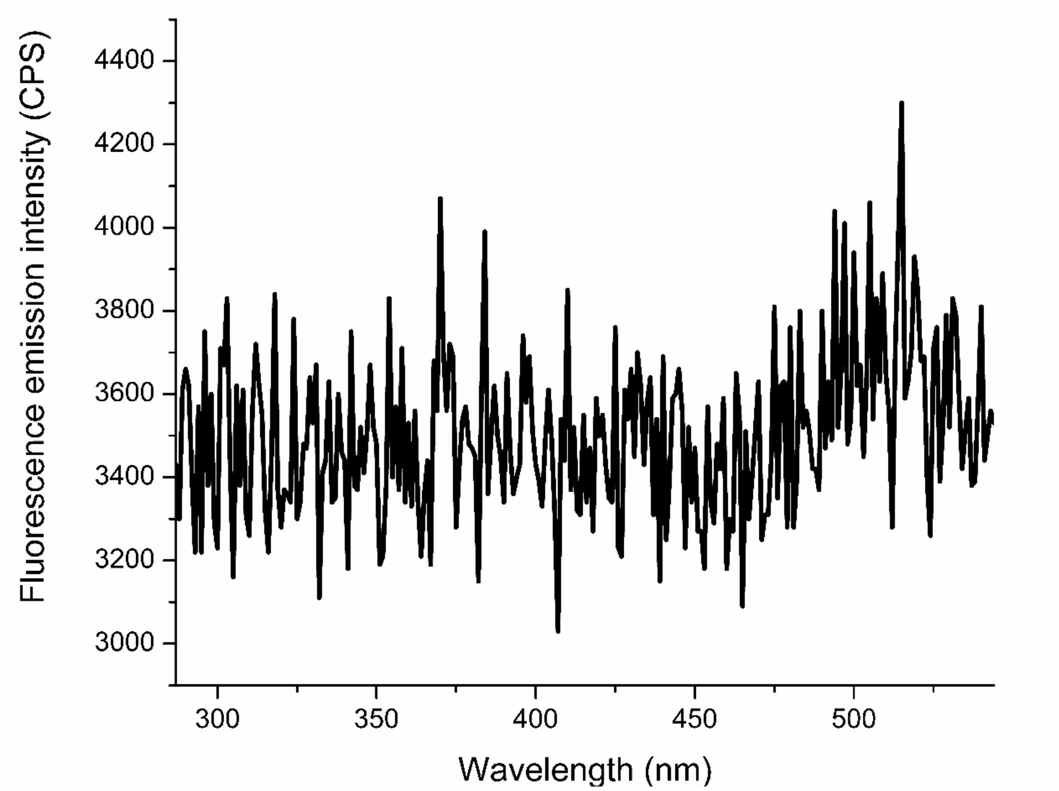

Figure S3. Fluorescence emission spectra of Na2Fl excited at 277 nm.

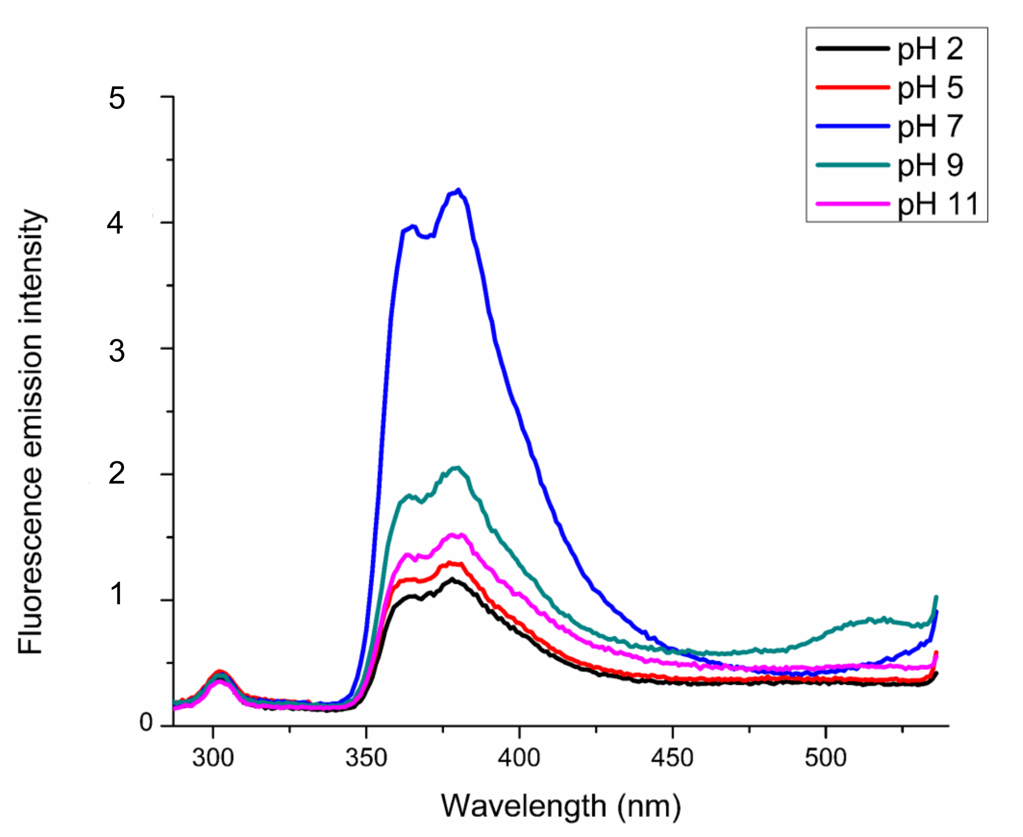

Figure S4. Emission of CII at varying pH values when excited at 277 nm.

pH buffers for adsorption studies

pH 2:

Combine 25 ml 0.2 M glycine and 22.0 ml HCl and dilute to 100 ml with DI

pH 4:

Combine the following proportions of 164 mL 0.1 M acetic acid and 36 mL 0.1 M sodium acetate

pH 6:

Combine 32.1 mL 0.2 M dibasic sodium phosphate; 17.9 mL 0.1 M citric acid

pH 8:

0.1 M PBS

pH 10:

Combine 25 ml 0.2 M glycine stock solution with 22.75 ml 0.2 M NaOH and dilute with DI to make a 100 ml solution
